# Editors’ pick: codeine toxicity prediction in young infants – genotype the mothers

**DOI:** 10.1186/2041-2223-3-24

**Published:** 2012-11-27

**Authors:** Antti Sajantila

**Affiliations:** 1 Department of Forensic Medicine, Hjelt Institute, University of Helsinki, Helsinki, Finland

## 

Codeine is a common analgesic used for postpartum pain in breastfeeding mothers. However, adverse reactions can happen even at low dosage, and evaluation of clinical risk factors for codeine toxicity, such as codeine dosage and administration time, are not sufficient alone to prevent such unfavorable events. A factor underlying this phenomenon is genetically determined variation in codeine response, which may range from poor analgesia to life-threatening central nervous system (CNS) depression. In 2006 an alarming case was published in *The Lancet*[1], describing a case of a barely two-week-old neonate who died from morphine poisoning by exposure to the drug through his mother’s milk. The high morphine concentration in the baby was found to be caused by the mother’s ultrarapid metabolizer (UM) phenotype due to a *CYP2D6* gene duplication. This case led health officials in several countries to issue warnings of codeine use for nursing mothers. Meanwhile, the scientific community has made an effort to find predictive markers to increase the effectiveness and safety of codeine usage. In a new study, published in April 2012 in *Clinical Pharmacology & Therapeutics*, Sistonen *et al.*[2]. report new data. The authors addressed the codeine toxicity in infants by genotyping their nursing mothers for codeine and morphine metabolizing enzymes CYP2D6 and UGT2B7, respectively, as well as morphine transporting P-glycoproteine encoding *ABCB1*, opioid receptor *OPRM1*, and mu-receptor interacting *COMT* genes. They further collected comprehensive clinical information from 111 mothers belonging to a Motherisk program in Canada, of which 26 (23%) reported CNS depression in their babies and 37 (33%) indicated typical codeine adverse reactions in themselves. When clinical data from 26 infants with CNS depression were compared with 85 mothers with asymptomatic babies two parameters were significantly different: mothers with symptomatic babies were 2.8 years younger and they consumed 0.27 mg/kg more codeine *per* day. Interestingly, *CYP2D6* genotype prediction of ultrarapid enzyme activity was significantly higher in the cases than controls (11.5% vs 2.4%), conferring an odds ratio (OR) of 16.5 when compared with the poor metabolizers. Similarly, three single nucleotide poilymorphisms (SNPs) in the *ABCB1* gene had significantly higher allele and genotype frequencies in cases vs controls, conferring ORs ranging from 5.45 to 6.63. Furthermore, the authors showed additive value of genotyping. The predictive model for codeine-induced CNS depression improved for infants (P = 0.028), mothers (P = 0.029) and both (P = 0.008). It is noteworthy that most of the neonates were under two weeks old, whose UGTB27 enzyme capacity is still reduced at that age. The genetic pathway leading to CNS depression in these cases is thought to be as follows: increased CYP2D6 activity leads to abnormal conversion of codeine to morphine. The morphine is then accumulated in the absence of UGTB27 activity in these neonates, and the CNS depression is enhanced by decreased activity of ABCB1, which mediates the cellular efflux of morphine at the blood–brain barrier. Altogether, the major importance of the findings of Sistonen *et al*. is that they have designed a novel set of SNPs in two genes (*CYP2D6* and *ABCB1*), which at least in the studied samples predicts over 80% of the codeine-induced CNS depression in nursing mothers and their infants. When combined with the known clinical risk factors, almost 90% of the risk cases can be predicted with a sensitivity of 80% and specificity of 87%. This study is the first to investigate the role of genetic variation in the morphine pathway related to codeine toxicity, and is clearly a promising step forwards in drug safety.
